# Study on the excess seeds removing performance of a potato precision seed metering device

**DOI:** 10.1038/s41598-024-79635-1

**Published:** 2024-11-13

**Authors:** Jiarui Wang, Min Liao, Chao Su, Rui Chen, Hailong Xia, Junju Li, Chenyang Qiao

**Affiliations:** 1 Institute of Modern Agricultural Equipment, Xihua University, Chengdu, 610039 China; 2 School of Mechanical Engineering, Xihua University, Chengdu, 610039 China

**Keywords:** Engineering, Mechanical engineering

## Abstract

In the potato mechanical planting industry, the high multi-seeding rate and miss-seeding rate are problems that exist in many potato precision seed metering devices. The main potato seeds in China are cut potatoes with different shapes and sizes. However, there is a lack of research on precision seeding technology for cut potatoes, and the adaptability of many existing potato seeding devices on cut potato is poor. The mechanized precision seeding of potato can effectively reduce labor consumption, improve the seeding effect (decrease multi-seeding rate and miss-seeding rate), improve the improve production of potato per acre and improve the quality commodity potatoes. In response to the above issues, a precision seed metering device with novel scoops, which can adapt to various sizes and shapes of cut potatoes, was developed. In this study, the different seed collecting scenarios (SCSs) were constructed. Through the statics and kinematic analysis, the excess seeds removing (ESR) process was divided in to three areas. The kinematic model of seed potato was established to study the influence of different SCSs and sprocket rotational speeds on the ESR performance, and preliminary determined the appropriate sprocket rotational speed range for ESR process. Then, the coupled simulation results of EDEM and RecurDyn revealed that different sprocket speeds and SCSs had a significant influence on the ESR performance. Finally, the bench tests and field tests were conducted to verify the actual performance of the precision seed metering device. The results showed that, with the increase of the sprocket rotational speed, the ESR performance of the device improved, the multi-seeding rate significantly decreased, but the miss-seeding rate increased. High sprocket rotational speed will remove more seed potatoes and cause high miss-seeding rate. The bench tests showed the appropriate sprocket rotational speed for medium and large potato was 35 r/ min. The field tests obtained the best qualified-seeding rate of 91.54%, the miss-seeding rate of 3.08% and the multi-seeding rate of 5.38%. This study can provide theoretical reference for the design of potato precision seed metering device.

## Introduction

Due to the well adaptability of potato to the environment, potato is one of the staple foods and grown in many countries^1,2^. And it is also the fourth largest crop which can increase incomes of farmers in China^3^. But in recent years, the mechanization level of potato seeding was about 20% in China^4^. So far, many researchers developed different types of potato planters^5^. The miss-seeding rate and multi seeding rate are crucial performance indicators of the potato seed metering device^6,7^. The mechanized precision seeding of potato can effectively reduce labor consumption, improve the seeding effect (decrease multi-seeding rate and miss-seeding rate), In the potato production, a high multi-seeding rate results in potatoes being too small in size and irregular in shape (commodity potatoes with bad quality), while a high miss-seeding rate leads to decreased potato production. Some researchers conduct studies on potato planters to reduce the miss-seeding rate and multi-seeding rate^8,9^. However, researchers seldom conducted study to analysis the adaptability of the seed metering device to cut potatoes (a kind of seed potato widely used in China). The existing planters mainly include chain-scoop seeding type and vacuum-seeding type^10,11^. Zhang et al.^11^ designed a novel potato planter with a combination metering device of vacuum seeding and scoop belt seeding, and studied the influence of seeding speed, cleaning-seed air amount and scoop aperture on the miss-seeding rate. Lü et al.^12^ designed an air suction potato planter, and studied the influence of device rotating speed and picking vacuum pressure on the miss-seeding rate and multi-seeding rate. But the influence of irregular shape of seed potato on the seeding performance is not studied. Some researchers have used EDEM to study the influence of factors on seeding performance of planter^13–15^. Cai et al.^16,17^ have used the discrete element method to analysis the motion of seed potato population in seeding process, and conducted seed collecting bench tests to verify the analysis. Qiu et al.^18^ used EDEM to optimize a precision seed-metering device. It is feasible to use this study method to analysis the adaptability of seed metering device to cut potato. Some researchers designed compensation system to reduce the miss-seeding rate^19,20^. Sun et al.^21^ designed a novel supplementary seeding device to reduce the miss-seeding rate. Qiu et al.^22^ designed a scoop-chain potato seed compensation system to reduce the miss-seeding rate. However, few researchers studied the way to decrease the multi-seeding rate and maintain a low miss-seeding rate for cut potatoes without any compensation system. Although a previous study develop a pickup finger to collect single global entire seed potatoes^23^, this device cannot effectively collect cut potatoes.

However, few researchers studied the adaptability of precision seed metering device to cut potatoes and the influence of SCS on the performance of ESR device. It is obvious that the motion characteristics of cut potatoes (with various sizes and shapes) are quite different from the normal entire seed potatoes and may cause to higher multi-seeding rate and miss-seeding rate during the seeding process. It is necessary to develop a potato seed metering device which can well adapt to cut potatoes and has low multi-seeding rate and miss-seeding rate. To address above problems, in this study, a precision seed metering device installed with novel scoops and compatible to cut potatoes was designed and analysed. The seed metering device can remove excess seed potatoes to reduce the multi-seeding rate and also can maintain a low miss-seeding rate. Through the theoretical analysis and numeric simulations, the suitable sprocket rotational speeds were obtained. The actual ESR performance of the precision seed metering device was verified by the bench tests and field tests. This study developed a precision seed metering device effectively adapt to cut potatoes, also can provide a theoretical and practical reference for the research on potato mechanized planting equipment.

## Materials and methods

### Structure and working principle of precision seed metering device

**Fig. 1:**
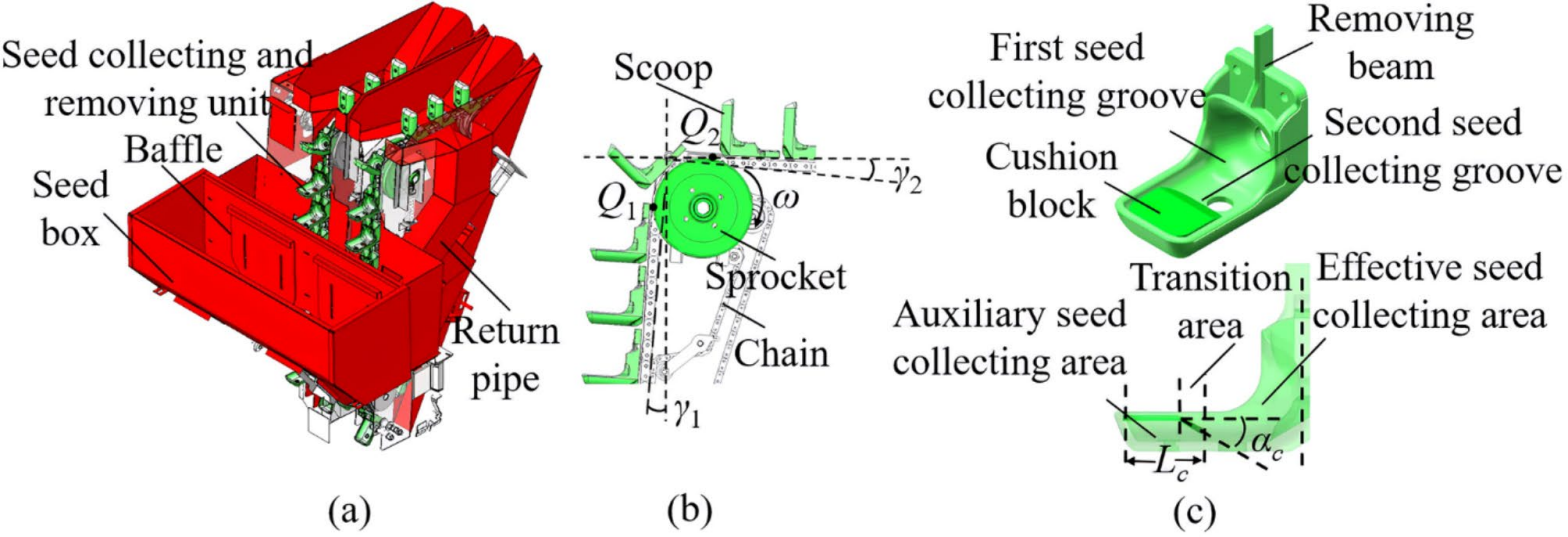
Structural diagram of precision seed metering device. (**a**) Overall structure, (**b**) collecting and excess seeds removing unit, (**c**) structure of scoop.

The structural diagram of precision seed metering device is shown in Fig. 1. The overall precision potato seed metering device is mainly composed of seed box, baffles, seed collecting and removing units and return pipes, as shown in Fig. 1a. The seed collecting and removing units are mainly composed of chains, scoops, sprockets, as shown in Fig. 1b. The *γ*_1_ represents the tilting angle of chains in seed collecting process relate to the vertical line, which is 5°. The *γ*_2_ represents the tilting angle of chains after ESR process relate to the horizontal line, which is 5°. A novel scoop is shown in Fig. 1c. The seed scoops are installed on the chains. The removing beam on the scoop can avoid seed potatoes remain on the chain. According the ability of storing seed potatoes in different process, the scoop is divided into first and second seed collecting groove. The second seed collecting groove is bigger than the first seed collecting groove and can directly collect enough seed potatoes in seed box (decreasing the miss-seeding rate). Theoretical, the first seed collecting groove can storage only one seed potato in it. A detachable cushion block is installed on the scoop, which has a flat surface in front and a tilting surface in rear with 30° tilting angle *α*_*c*_, and the length of cushion block is *L*_*c*_. According to the function in seed collecting process, the scoop is divided into auxiliary seed collecting area, transition area, and effective seed collecting area. The cushion block flat surface is auxiliary seed collecting area. The tilting surface of the cushion block is the transition area. The second seed collecting area is the effective seed collecting area.

The working process of the precision seed metering device includes 3 steps. Step 1, the sprockets rotate clockwise at the rotational speed of *ω*, and the scoops on chains will move upward to collect seed potatoes. Step 2, the scoops carried with seed potatoes will go up, start to flip at point *Q*_1_, and end the flip at point *Q*_2_. During the flipping process, the scoops will rotate clockwise for about 90° around the center of the sprocket. After flipping, theoretically, only one seed potato will remain in a scoop, and the excess seed potatoes will fall out of scoop. Step 3, the scoops carried with single seed potato continue to move to the seeding position. The removed excess seed potatoes will return to seed box via the guidance of return pipes. The flipping process is also called ESR process. Usually, the seed scoop will collect enough seed potato (more than one) to ensure a low miss-seeding rate. Then, the ESR process will remove the excess seed potatoes to ensure a low multi-seeding rate.

### Analysis of seed collecting scenarios

**Fig. 2:**
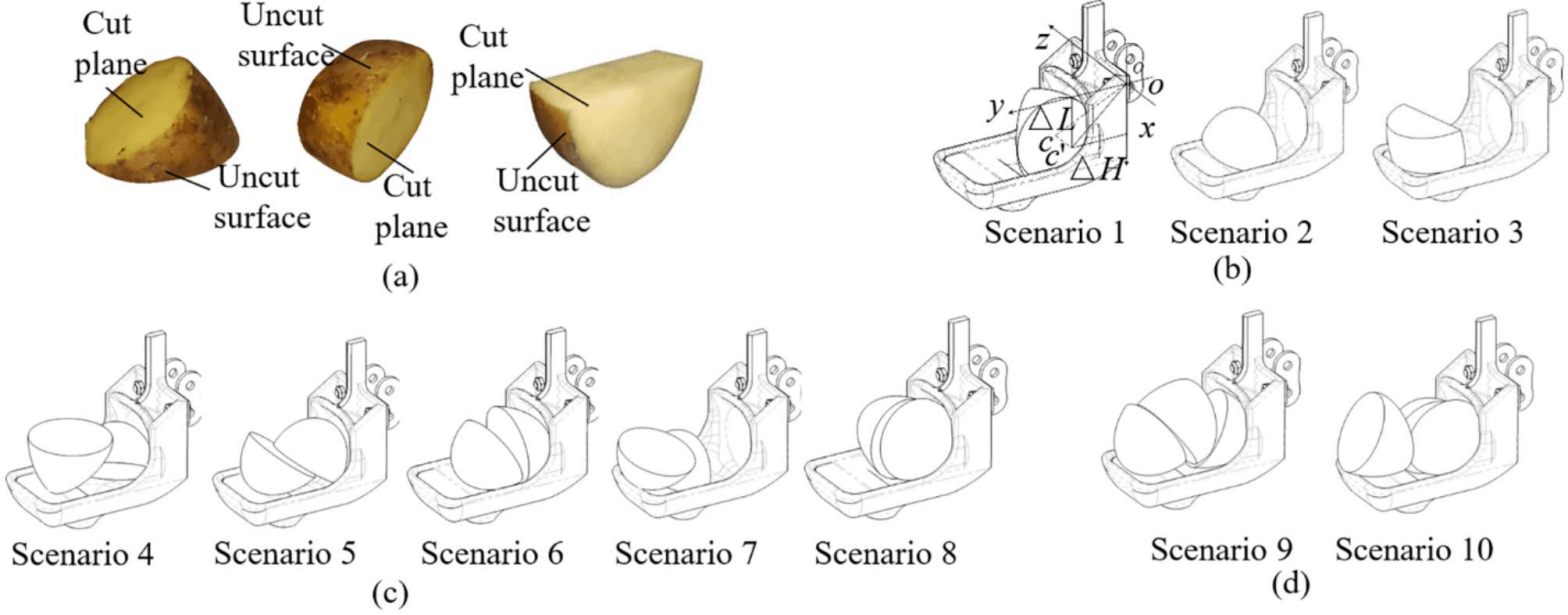
Analysis of single SCSs: (**a**) common cut potato shape; (**b**) schematic diagram of single SCSs; (**c**) schematic diagram of double SCSs; (**d**) schematic diagram of treble SCSs.

The ESR performance of the seed metering device can be different in different seed collecting scenarios. The analysis of seed collecting scenarios is a crucial step for conducting theoretical analysis. The shape of cut potatoes is various and can result in overlapping or big gaps in scoop, as shown in Fig. 2. In this study, the most common cut potatoes are divided into 3 types according to the different cut surface, as shown in Fig. 2a. The SCSs are divided into 3 types according to the number of seed potatoes in the scoop (single seed, double seeds and treble seeds), and these 3 SCSs were divided into 10 scenarios according to the posture of seed potatoes in the scoop, as shown in Fig. 2b–d.

1. Single seed. The analysis of single SCSs is shown in Fig. 2b. If a seed is within the effective seed collection area and protected by the scoop’s surroundings, no matter how the seed potatoes come into contact with the scoop, it can be regarded as scenario (1) If a single seed potato is lying on the cushion block surface and partly protected by the scoops surroundings, it can be regarded as scenario (2) The cut surface of seed potato is closely contacted with the cushion block tilting surface, and the seed potato is tilted at 30° angle, (the inclination angle of the cushion block surface is 30°). Since the static sliding angle of seed potato is obtained to be 27°^24^, the seed potato will slide to make contact with the bottom of second seed collecting groove. If the uncut surface of the seed potato contacts with the cushion block tilting surface, the seed potato is very unstable and prone to roll into the effective seed collecting area and become scenario 1 after the ESR action. If a single seed potato lies on the flat surface of the cushion block, regardless of how the seed potatoes contact with the scoop, it can be regarded as scenario (3) The seed potato is likely to roll when its uncut surface comes into contact with the cushion block after the ESR process.
2. Double seed. As shown in Fig. 2c, the scenarios 4 and 5 are similar, resembling a combination of scenario 1 and scenario 3, one seed potato in the effective seed collecting area and another in the auxiliary seed collecting area. In scenario 4, the contact face of two seed potatoes is uncut surface to uncut surface, with a large gap. The contact face of seed potato in auxiliary seed collecting area to scoop cushion block is uncut surface. There is a large gap between these two potatoes, which makes the seed potato in the auxiliary seed collecting area in an unstable status. Like scenario 1, no matter how the seed potato in effective seed collecting area contacts with the scoop, the seed potato is protected by the scoops surroundings and in a stable status (hard to be removed out of scoop by ESR process). In scenario 5, the contact face of two seed potatoes is cut surface to cut surface, with a gap smaller than scenario 4. The contact faces of seed potato in effective seed collecting area and auxiliary seed collecting area to scoop are both uncut surface. This contact method is more stable than scenario 4. The scenario 6 has two seed potatoes close to each other, one seed potato is in the effective seed collecting area and another is in the transition area. The contact face of two seed potatoes is cut surface to cut surface, and the gap is bigger than scenario 5. The contact face of two seed potatoes to the scoop is alike scenario 5. In scenario 7, resembling a combination of scenario 1 and scenario 2, one seed potatoes is in the transition area and another is in the auxiliary seed collecting area. The contact face between two seed potato is uncut surface to uncut surface, and the gap is large. The seed potato in the auxiliary seed collecting area can be easily removed out of scoop by ESR process, but another seed potato is hard to be removed out. In scenario 8, two seed potatoes are pressed together in the effective seed collecting area. The contact face between two seed potatoes is cut surface to cut surface, and the gape is very small. The contact faces of potatoes to scoop are both uncut surface. It is hard to remove these seed potatoes out of scoop by ESR process. However, the scoop is well designed and this scenario rarely occurred in our actual experiments. The occurrence rate of scenario 8 also can be reduced by properly select cushion block and prepare cut potatoes with suitable sizes.
3. Treble seed. The most common SCSs of three seed potatoes are mainly divided into two scenarios as shown in Fig. 2d. The scenario 9 filled all areas with seed potatoes. The contact face between the seed potato in effective seed collecting area and the seed potato in transition area is cut surface to uncut surface, with a big gap. The seed potato on the cushion block flat plane is in an unstable status and easy to fall out of scoop. In this scenario, usually, only the seed potato in the effective seed collecting area can be remained in the seed scoop after the ESR process. The scenario 10 is similar to scenario 8 in double SCS. Two seed potatoes are in the effective seed collecting area, and the other seed potato is in the auxiliary seed collecting area in an unstable status. The contact face of the two potatoes closest to the effective seed collecting area is cut surface to cut surface, and the gap of these two seed potatoes is very small. The ESR process can remove the cut potato in the auxiliary seed collecting area. The occurrence rate of scenario 10 also can be reduced by properly select cushion block and prepare cut potatoes with suitable sizes.

### Theoretical analysis of excess seeds removing (ESR) process

In this study, the centroid of potato is taken as the reference for analysis the motion status of seed potatoes. And an orthogonal Cartesian coordinate system was constructed for determining the potato centroid, as shown in Fig. 2b. In the coordinate system, point *o* (top-right corner of the scoop’s back) is the origin point, the *x*- axes is downward along the scoop’s back, the *y-*axis is perpendicular to the scoop’s back, and *z*-axis is perpendicular to the *xoy* plane. Supposing the seed potato centroid position and its reflection on *xoy* plane are *c* and *c*’ respectively, the distances of point *c*’ to the *x*-axis and *y*-axis are △*H* and △*L* respectively. After calculation, the values of △*H* in scenario1, 2, and 3 are 37 mm, 27 mm and 84 mm, respectively. The average values of △*L* in scenario1, 2, and 3 are 35 mm, 30 mm and 24 mm, respectively. In this section, the movement of seed potatoes in different SCS during this process was analyzed.

1. Single seed collecting. The ESR process of collecting scenario 2 and 3 are divided into 3 major areas according to the statics analysis of seed potato, as shown in Figs. 3 and 4. The area between line *OA* and *OB*, *OB* and *OC*, *OC* and *OD* are few sliding area, sliding or parabolic area and main parabolic area in theoretical analysis, respectively. The range of each area depends on the SCS and sprocket rotational speed. In this section, to preliminary study the suitable range of sprocket rotational speed for ESR process, the sliding movements were ignored.
  **Fig. 3:**
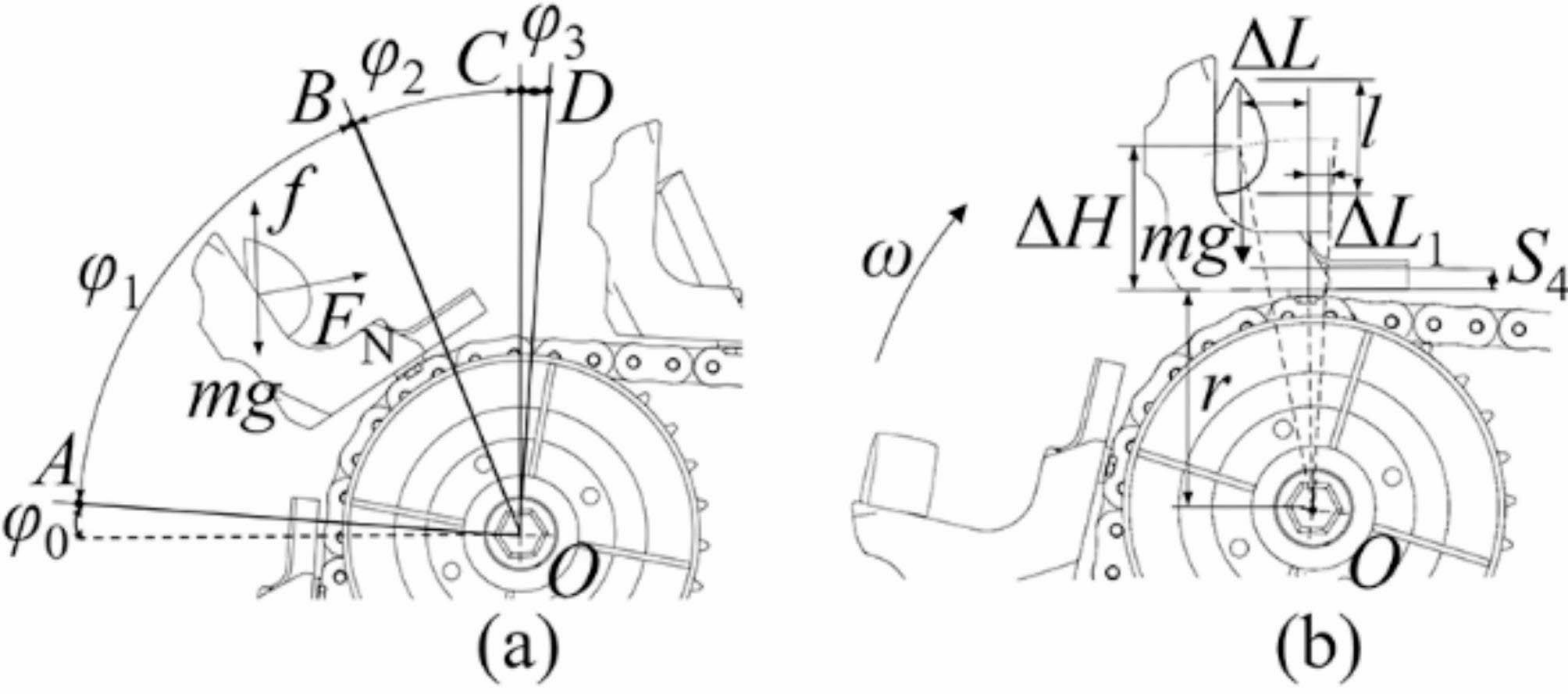
Schematic diagram of flipping analysis in scenario 2: (**a**) different division of flipping area, (**b**) analysis of flipping.
  **Fig. 4:**
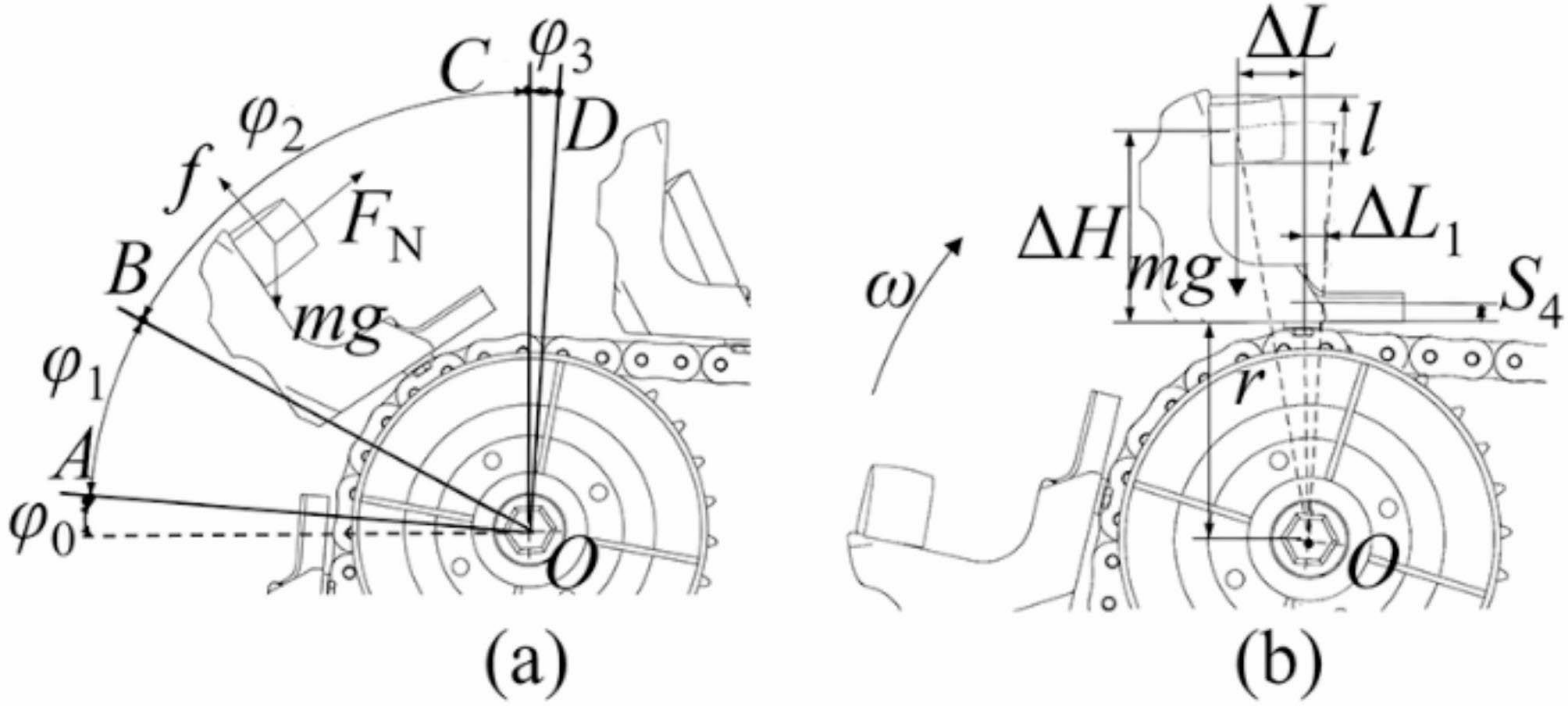
Schematic diagram of flipping analysis in scenario 3. (**a**) Different division of flipping area, (**b**) analysis of flipping.
  In scenario 1, because the seed potato is in the effective seed collecting area and well protected by the surroundings of the scoop, it will quickly move into the first seed collecting groove with little displacements after flipping action. Thus, it is hard for the seed potato in effective seed collecting area to fall out of scoop by simple flipping action, and the scenario 1 needs no analysis. In scenario 2, for the seed potato is on the cushion block rear surface, the seed potato is tilted at 30° in the scoop (*α*_*c*_ = 30°). Taking the installation position of the scoop on the chain as the reference point for its movement analysis, and the schematic diagram of flipping analysis in scenario 2 is shown in Fig. 3. The supporting force *F*_*N*_ is vertical to the cushion block tilting surface and upward, the friction force *f* on the seed potatoes is along the cushion block tilting surface and upward. The direction of gravity *mg* is vertical down. The flipping process starts when the scoop reaches line *OA*. In this moment, due to the tilting angle of chains *γ*_1_ is 5° (as shown in Fig. 1b), the *φ*_0_ is about 5°. The tilting angle of seed potato in this status is about 35°, but the seed potato contacts with the bottom of the second seed collecting groove and slightly slides. When scoop reaches line *OB* with about 60° scoop tilting angle, and the *φ*_1_ is about 55°, the actual tilting angle of seed potato reaches almost 90° and in a vertical status. The seed potato in this status is about to do parabolic motion, but it quickly slides to make contact with the bottom of second seed collecting groove, or may moves into the second seed collecting groove and become scenario 1. When the scoop reaches line *OC* and with 90° tilting angle, the seed potato starts to do parabolic motion again and accelerates under the force of gravity, and the *φ*_2_ is about 30°. Finally, the flipping action completes when the scoop reaches line *OD* with about 95° scoop tilting angle, and the *φ*_3_ is about 5°. The seed potato moves nearly horizontal after line *OD*. During the entire ESR process in scenario 2, the parabolic movements are very few compared to sliding movements.
  In scenario 2, since the sliding area is bigger than parabolic area, the relative displacements of seed potato to scoop caused by sliding movements are greater than those caused by parabolic movements. And the sliding movements make the seed potato stably close to the first seed scoop and become harder to be removed out of scoop. To obtain the maximum linear speed of chain to avoid the seed potato in this scenario fall out of scoop, assuming the sliding movements of seed potato from line *OA* to line *OC* are ignored and the chain motion is considered uniform, the kinematics analysis was conducted on the seed potato that just reaches line *OC*, as shown in Fig. 3b. The seed potato’s movement is simplified to parabolic, while it rotates and moves horizontally within the scoop. To ensure the potato stays within the first seed collecting groove after ESR process, the movements of seed potatoes should meet Eq. (1).
  1 $$\left\{ \begin{gathered} {v_s}=(r+\Delta H){\omega _s}/2\pi \hfill \\ \Delta H - {S_4} - \frac{1}{2}l - \frac{1}{2}gt_{{}}^{2}=0 \hfill \\ {v_s}t - (\Delta L+\Delta {L_1})<0 \hfill \\ \end{gathered} \right.$$
  Through the analysis of Eq. (1), to ensure the seed potato in transition area will fall into the first seed collecting groove, the maximum speed of chain *v*_*s*_ should meet Eq. (2).
  2 $${v_s}<(\Delta L+\Delta {L_1})\sqrt {\frac{g}{{2\Delta H - 2{S_4} - l}}}$$
  Where *v*_*s*_ is the maximum linear speed of chain at line *OC*, Δ*L*_1_ is the increment of horizontal displacement at the edge of the scoop (= 11 mm), *ω* is angular velocity of sprocket, *S*_4_ is the distance from the bottom of first seed collecting groove to the base of the scoop (= 25 mm), *l* is average length of seed potatoes (= 54 mm), *r* is rotation radius of the scoop base (= 97 mm), and *g* is gravitational acceleration. And in line *OC*, the *t* is dropping time of seed potatoes, the Δ*H* is 67 mm, and Δ*L* is 30 mm.
  Tacking the parameters in to calculation, we can obtain the *t* = 0.05 s, *v*_*s*_<0.71 m/s, that is, *ω*_*s*_ < 42 r/min. Thus, the sprocket rotational speed *ω*_*s*_ should be smaller than 42 r/min to prevent the seed potato in scenario 2 from fall out of the scoop in ESR process.
  The scenario 3 is rare in actual seeding work, but the scenario 4, 5,7 and 9 is very common in actual seeding process with a seed potato lying on the auxiliary seed collecting area. Thus, it is necessary to analysis how to remove the seed potatoes in the auxiliary area. The schematic diagram of flipping analysis in scenario 3 is shown in Fig. 4. The supporting force *F*_*N*_ is vertical to the cushion block tilting surface and upward, the friction force *f* on the seed potatoes is along the cushion block tilting surface and upward. The direction of gravity *mg* is vertical down. The line *OA* is the start position of ESR process in scenario 3, and the scoop tipping angle in this status is 5°, and the angle *φ*_0_ is 5°. At line *OB*, the scoop tipping angle increased to 30° and bigger than the critical sliding angle of seed potato, and the angle *φ*_1_ is about 25°. If the contact face of seed potato to cushion block is uncut surface, the seed potato will start to toward the effective seed collecting area from line *OA* to line *OB*. If not, the seed potato is about to slide after passing through the line *OB*. From the line *OB* to line *OC*, the potato starts to do sliding movements along an arc trajectory, but not fast enough to reach the effective seed collecting area. At line *OC*, the scoop is vertical, and the angle *φ*_2_ is about 60°. From line *OC* to line *OD*, the seed potato shows a parabolic motion before dropping quickly under gravity. However, the area from line *OC* to line *OD* is small, with an angle of *φ*_3_, and the seed potato’s centroid is near the edge of the first seed collecting groove and further from the bottom of the scoop, making it easier for the seed potato to be removed out of scoop. The ESR process ends at line *OD* with about 95° scoop tilting angle. The seed potato moves nearly horizontal after line *OD*. When comparing the ESR process for single scenario 2, the sliding movements in scenario 3 is later and smaller than scenario 2, and the parabolic motion in scenario 3 is later than scenario 2. The scenario 2 allows seed potato for more movements toward the first seed collecting groove before it is thrown out in a parabolic motion, which moves the seed potato’s centroid closer to the bottom of the scoop. And in scenario 3, the displacements of seed potato relative to the scoop caused by the parabolic movement displacements are bigger than those caused by sliding movements. Thus, the scenario 3 is easier to remove the seed potato than scenario 2.
  To determining the minimum chain speed for removing the excess seed potato in auxiliary seed collecting area, the chain motion is considered uniform, the kinematics analysis was conducted on the seed potato that just reaches line *OC*, as shown in Fig. 4b. The movement of seed potato in this status is regard as only parabolic motion. For effectively removing excess seeds, the centroid of seed potato must extend past the first collection groove’s edge after flipping. The relative horizontal displacement of the potato seed compared to the scoop should be more than Δ*L*, but the vertical displacement must be less than the difference between Δ*H* and *S*_4_. That is, the motion of seed potato should meet Eq. (3).
  3 $$\left\{ {\begin{array}{*{20}l} {v_{d} = (r + \Delta H)\omega _{d} /2\pi } \hfill \\ {\Delta H - S_{4} - \frac{1}{2}l - \frac{1}{2}gt^{2} = 0} \hfill \\ {v_{d} t - (\Delta L + \Delta L_{1} )0} \hfill \\ \end{array} } \right.$$
  Supposing the horizontal movement increment Δ*L*_1_ is 11 mm. The minimum chain’s speed *v*_*d*_ to remove the seed potatoes in auxiliary collecting area should meet Eq. (4):
  4 $${v_d}>(\Delta L+\Delta {L_1})\sqrt {\frac{g}{{2\Delta H - 2{S_4} - l}}}$$
  When the seed potato is at the auxiliary collecting area’s far end. In line *OC*, the Δ*H* is 84 mm, and the Δ*L* is 24 mm. After calculation of Eq. (4), we can obtain *v*_*d*_>0.43 m/s, that is, *ω*_*d*_ > 22.9 r/min.
  Thus, to throw the seed potatoes in the auxiliary seed collecting area (like scenario 3) out of scoop, the sprocket rotational speed should bigger than 22.9 r/min.
2. Double and treble seed: In ESR process, taking the scenario 4 and 5 as example, every scenario has one seed potato in the auxiliary seed collecting area and another in the effective seed collecting area. The seed potatoes in the effective seed collecting area are stable and hard to being removed. The ESR process need to remove the seed potato in the auxiliary seed collecting area. The flipping line speed of the seed potatoes in the transition area is higher than in the effective seed collecting area when the scoop is vertical. There is relative movement between the upper and lower layers of the seed potatoes. The statics analysis of seed potatoes in double SCS was conducted. As shown in Fig. 5, an orthogonal Cartesian coordinate system based on the centroid of the upper layer of seed potatoes was established. The *x*-axis direction is rightwards along the contact surface of the upper and lower layers of seed potato, and the *y*-axis direction is perpendicular to the contact surface of the upper and lower layer seed potatoes. The upper layer of seed potatoes slides relatively to the lower layer of seed potatoes during ESR process. The kinetic energy of the upper layer seed potatoes overcome the work done by friction and gravity. The supporting force *F*_*N*_ is along the *y-*axis direction, and the friction force *f* on the seed potatoes is negative along the *x*-axis direction. The direction of gravity *mg* is vertical down. From the analysis above, the Eq. (5) can be obtained as:
  5 $$\left\{ \begin{gathered} \frac{1}{2}mv_{m}^{2} - (f+G\cos \delta ){L_x} - mgh>0 \hfill \\ v_{m}^{{}}=v_{n}^{{}} - v_{k}^{{}} \hfill \\ F_{N}^{{}}=G\sin \delta \hfill \\ f=\mu F_{N}^{{}} \hfill \\ \end{gathered} \right.$$
  Where *v*_*m*_ is relative speed of upper and lower layer seed potatoes, *v*_*n*_ is upper layer seed potato linear speed. *v*_*k*_ is linear velocity of lower layer seed potatoes, *L*_*x*_ is movement displacement of upper layer seed potatoes relative to lower layer seed potatoes along *x*-axis, *δ* is the angle between the contact slope of the seed potato and the direction of gravity, *h* is vertical increment of seed potato centroid.
  **Fig. 5:**
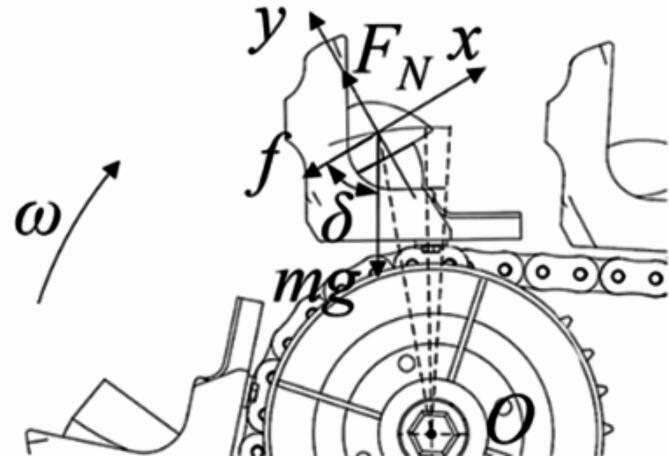
Analysis of ESR process for double and treble-seed collecting.
  According to Eq. (5) and Fig. 5, it can be seen that the difficulty of removing excess seeds in scenario 4, 5 and 6 is related to the speed of the chain (or sprocket rotational speed) and the contact scenario of the upper and lower layers of seed potatoes. The faster the sprocket rotational speed, the greater the relative speed *v*_*m*_ of the upper and lower layers of seed potatoes. And the upper layer of seed potatoes gains more kinetic energy to overcome frictional resistance and flip out of scoop. The effect of seed potato gravity in the process of flipping and removing seeds is determined by the angle *δ* between the contact surface of the upper and lower layer of seed potatoes and the direction of gravity. When the scenario 4 is flipped to the throwing position, the component of gravity along the *x*-axis is in the positive direction of the *x*-axis, and gravity is the driving force for flipping and removing excess seeds. Thus, the scenario 4 is easier to remove excess seed potatoes than scenario 6, similarly, the scenario 6 is easier than scenario 5 (As shown in Fig. 3). The contact scenario of the upper and lower layers of seed potatoes is complex, diverse, and uncontrollable, so the rotational speed of the sprocket is an important factor affecting the success of excess seed removing. In conclusion, it is easy for scenario 4 to remove excess seed potatoes. The scenario 5 and scenario needs faster sprocket rotational speed to remove excess seed potatoes.
  In scenario 7, the seed potatoes are located in the auxiliary seed collecting area and the transition area, respectively, which is a composite scenario of scenario 2 and 3. From the analysis of single SCSs, it can be seen that, the transition area seed potatoes start to slide before the auxiliary seed collecting area during the ESR process. When the speed of the sprocket is within the theoretical limit speed, the effective seed collecting area is occupied by the transition area seed potato firstly, leaving no space for the seed potatoes in auxiliary seed collecting area. This causes the excess seed potato collide with the seed potato in the effective seed collecting area and successfully fell out of scoop. The scenario 7 can effectively remove the excess potato on the auxiliary seed collecting area by adjusting the sprocket to a proper rotational speed. The rotational speed of sprocket should faster than *ω*_*s*_ to remove the seed potatoes in auxiliary seed collecting area. In scenario 8, both seed potatoes are in the effective seed collecting area and squeezed against each other, and the excess seed potatoes are hard to be removed out of scoop. Since the designed scoop cannot contain two seed potatoes in one effective seed collecting area, it prevents scenario 8 from occurring during the actual seeding process. Scenario 8 is considered as failure SCS and needs no analysis. We also can effectively reduce the occurrence of scenario 8 by selecting a proper cushion block size and preparing cut potatoes with uniform and suitable size. The treble SCSs combine elements of both single and double SCSs. The ESR process of scenario 9 is similar to double SCSs 4, 5, and 6, and the process is largely influenced by the speed of the sprocket. The scenario 9 can also effectively remove the excess seed potatoes by adjusting the sprocket to a suitable rotational speed. The scenario 10 is similar to compound scenario 8 in double SCSs and scenario 3 in single SCSs. It can only remove seed potatoes in the auxiliary seed collecting area by adjusting the sprocket rotational speed, and whether the other excess seeds can be removed is uncertain. Scenario 10 is also considered as a failure SCS and needs no analysis. The occurrence of the scenario 10 also can be effectively decreased by selecting a proper cushion block and preparing cut potatoes with uniform and suitable size.
  Therefore, in the double and treble SCS, the excess seed potatoes in the auxiliary seed collecting area can be removed out of scoop if the sprocket rotational speed is within 22.9 r/min-42 r/min. The faster the sprocket rotational speed, the more seed potatoes can be removed out of scoop.

### Construction of numeric simulation of excess seeds removing (ESR) process

**Fig. 6:**
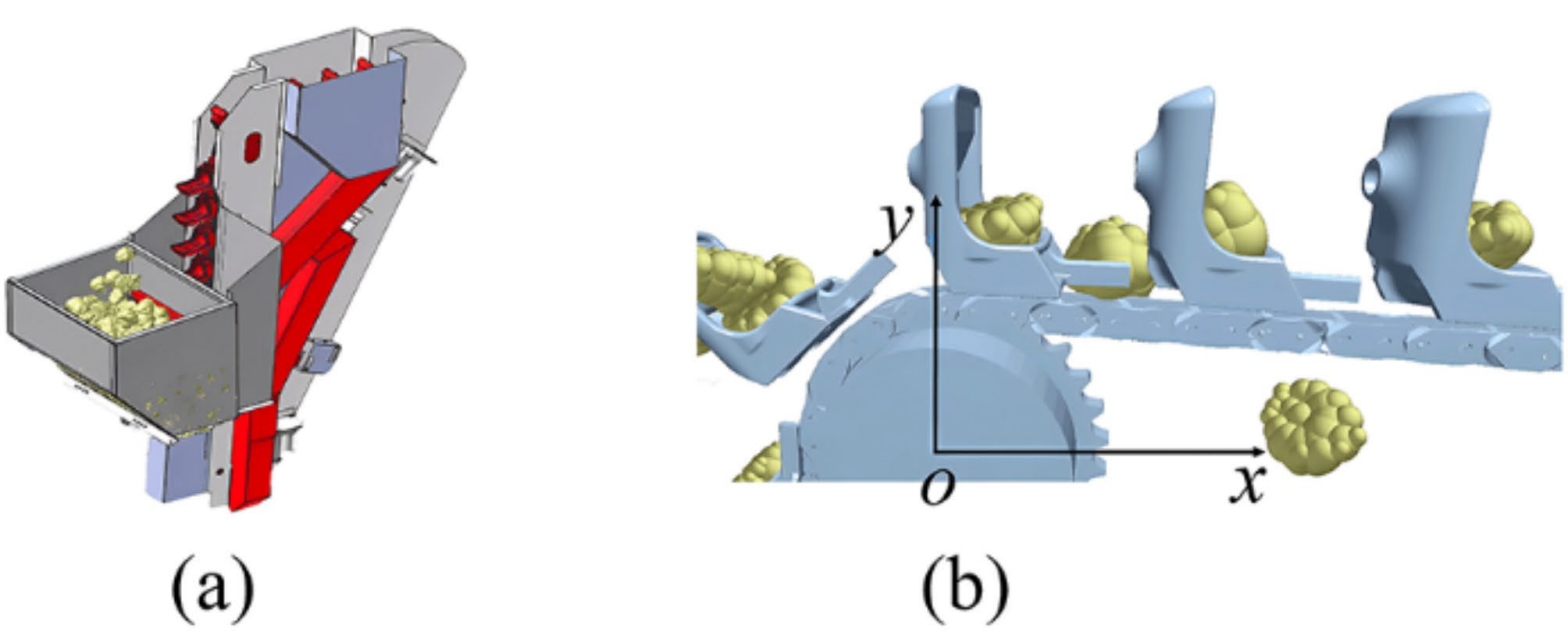
Simulation model. (**a**) Overall model, (**b**) construction of ESR process coordinate system.

A simulation was conducted to study the influence of SCSs and sprocket rotational speed on the trajectory of seeds during the ESR process. The appropriate sprocket rotational speed of ESR was obtained through simulation. The coupling simulation was constructed in RecurDyn and EDEM. The coupling of two software is widely used in agricultural analysis^25,26^. According to the above analysis, sprocket rotational speed of 25 r/min, 30 r/min, 35 r/min, 40 r/min, 45 r/min and 50 r/min were selected in simulation. The key parameters of seed potato and structure steel were determined by the previous study^24^. In the setting of simulation parameter, the Poisson’s ratio of cut potatoes was 0.48, the shear modulus of cut potatoes was 1.34*10^− 4^ MPa, the density of cut potato was 1048 kg/m^3^, the Poisson’s ratio of Q235 steel was 0.28 MPa, the shear modulus of steel was 7*10^4^ MPa, the density of Q235 was 7800 kg/m^3^, the static friction coefficient between cut potatoes and steel parts was 0.554, the coefficient of static friction between seed potatoes was 0.38, the dynamic friction coefficient between seed potatoes was 0.026, the collision recovery coefficient between cut potatoes and steel parts was 0.71 and the coefficient of restitution between different cut potatoes was 0.79. The constructed simulation model is shown in Fig. 6. The overall model is shown in Fig. 6a, with one seed collecting and removing unit installed on seed metering device. The seed position data during simulation was tracked. Supposing the sprocket’s rotation center is the origin point, with the upper direction and horizontal right direction being the positive direction of *y-*axis and *x*-axis respectively, an orthogonal Cartesian coordinate system was constructed for analysis the trajectory of potato centroid during ESR process, as shown in Fig. 6b.

### Preparation of bench and field tests

**Fig. 7:**
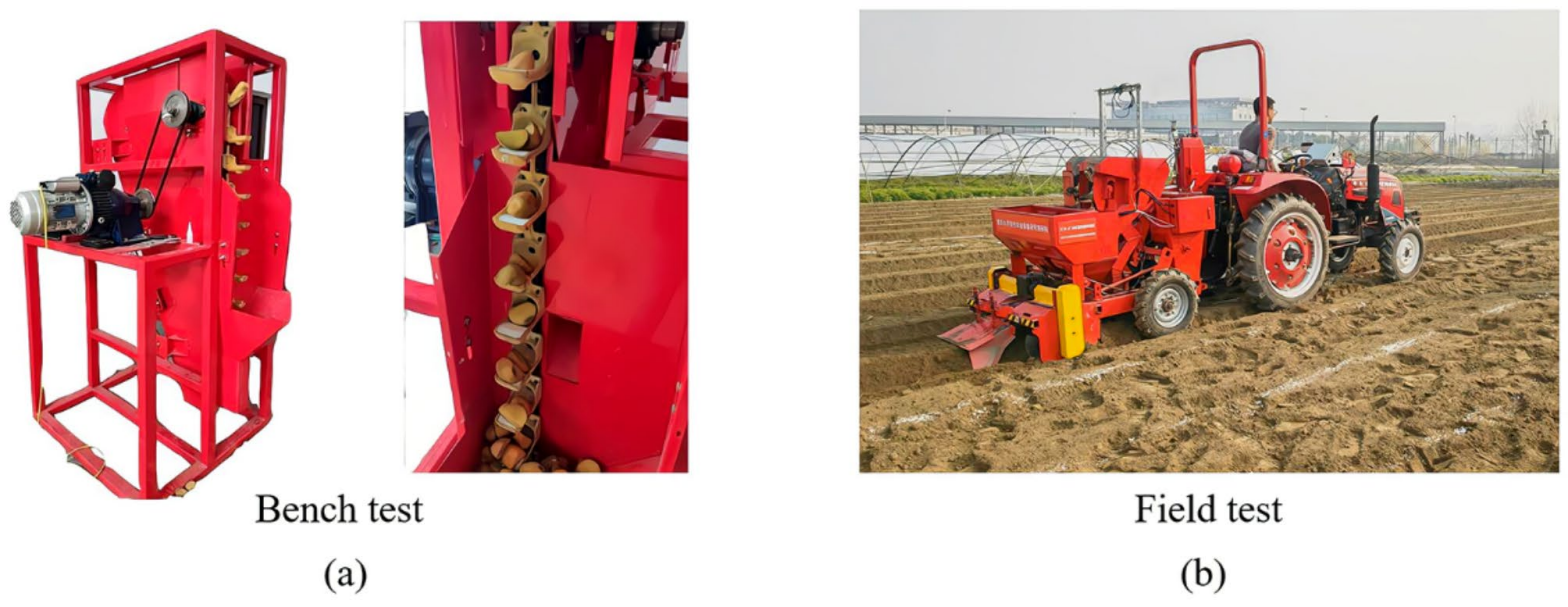
Bench tests and field tests. (**a**) Bench test, (**b**) field test.

To verify the actual performance of precision seed metering device, the bench tests and field tests were conducted, as shown in Fig. 7.

1. Bench tests. The bench tests were conducted based on a manufactured precision seed metering device testing bench, as shown in Fig. 9a. In order to verify the adaptability of the precision seed metering device to different sizes of seed potatoes, three group tests were designed and conducted. Three typical cut potato were prepared: large seed potatoes B3 (above 45 g), medium seed potatoes B2 (35 g ~ 45 g), small seed potatoes B1 (below 35 g). Three kinds of designed cushion blocks adapt to these cut potatoes were used in the tests: small cushion blocks C1 (with 40 mm length), medium cushion blocks C2 (with 50 mm length) and large cushion blocks C3 (with 60 mm length). And 3 levels of sprocket rotational speeds A1 (= 35 r/min), A2 (= 40 r/min) and A3 (= 45 r/min) were tested.
  The “DT2234C laser type tachometer” was used to adjust the rotational speed of the sprocket. The data collection process and statistical methods used to evaluate the trail indicators in bench tests are referred to a standard “GB/T 6973 − 2005 Testing methods of single seed drills (precision drills)”^27^. After the ESR process, the scoop carries no seed, a single seed, or more than one seed, representing miss-seeding, qualified-seeding, and multi-seeding, respectively. When a scoop collects more than one seed, if only one potato remains in the scoop after the ESR process, it is considered as qualified ESR. The qualification rate of ESR was calculated by dividing the number of scoops with only one seed left after ESR by the total number of scoops with more than one seed. In the bench test, 100 scoops were tested in every test group. Each test group was repeated for five times. To verify the actual seed removing performance of the ESR performance, the miss-collecting rate *P*_1_ and multi-collecting rate *P*_2_ before ESR process was also recorded. The calculation method to obtain the qualified-ESR rate *P*_3_, qualified-seeding rate *A*, multi-seeding rate *D* and miss-seeding rate *M* is shown in Eq. (6):
  6 $$\left\{ \begin{gathered} {P_1}=\frac{{{n_1}}}{N} \times 100\% \hfill \\ {P_2}=\frac{{{n_2}}}{N} \times 100\% \hfill \\ {P_3}=\frac{{{n_4}}}{{{n_1}+{n_3}}} \times 100\% \hfill \\ A=\frac{{{n_4}}}{N} \times 100\% \hfill \\ D=\frac{{{n_5}}}{N} \times 100\% \hfill \\ M=\frac{{N - {n_4} - {n_5}}}{N} \times 100\% \hfill \\ \end{gathered} \right.$$
  Where *N* is the amount of tested scoop, *n*_1_ is the miss-collecting amount, *n*_2_ is the multi-collecting amount, *n*_3_ is the qualified ESR scoop amount, *n*_4_ is the amount of scoop carried with only one seed after the ESR process, *n*_5_ is the amount of scoop carried with more than one seed after the ESR process.
2. Field tests. To conduct the field test, a potato planter equipped with the precision seed metering device was hanged on a Huanghai Jinma-504 tractor (with a power output of 36.75 kW), as shown in Fig. 9b. The field test was conducted in the test field located in Shuangliu District, Chengdu, Sichuan Province. The entire field was tilled and the soil was loose. After tillage, the soil moisture content was 14.5%, and the soil firmness was 1.35 g/cm³. In the field tests, the medium seed potatoes and C2 cushion blocks were used, and the sprocket’s speed was set to 35 r/min. Based on the transmission ratio (plant spacing) requirement, we set the forward speed of potato planter to 0.6 m/s. The“DT2234B Laser velocimeter” was used to adjust the forward speed of the potato planter. Five consecutive seeding operations were carried out and results were recorded. The data collection process and statistical methods used to evaluate the trail indicators in field tests are referred to a standard “GB/T 6242 − 2006; Equipment for planting Potato planters Method of testing”^28^. Four consecutive seeding operations were carried out and results were recorded. In the testing process, the first and last 5 m of each soil ridge planted with seed potatoes were excluded due to unstable seeding. The middle 260 pits in the test were recorded and analysis. The test was repeated for five times. The theoretical placing spacing of this precision seed metering device is 22 cm. Supposing the distance between the centers of closest two planted potato seeds is *L*_*t*_. After seeding process, any *L*_*t*_ that exceeds 1.5 times of the theoretical planting spacing (*L*_*t*_>33 cm) is recorded as one missed-seeding. The *L*_*t*_ exceeds 2.5 times of the theoretical planting spacing (*L*_*t*_>55 cm) can is recorded as two miss-seeding. Similarly, 3.5 times for 3 miss-seeding. And any *L*_*t*_ that less than 0.5 times the theoretical planting spacing (11 cm) is recorded as one multi-seeding. A tape measure (with an accuracy of 1 mm) was used to measure the distance between potatoes. the Five groups were tested The total number of qualified-seeding, miss-seeding and multi-seeding were added together as the total number of pits. The seeds on the ridge were counted to assess the seeding quality.

## Results

### Numeric simulation

1. Single seed collecting. Figures 8, 9 and 10 showed how the trajectories of seed potato in single SCSs changed with different sprocket rotational speeds. The *x* and *y* refer to the displacement components of the trajectory of seed potato along the *x*-axis and *y*-axes, and the point *O* represents the rotation center of sprocket. The movement trajectories are divided according to the actual moving status of seed potatoes in the simulation.
  **Fig. 8:**
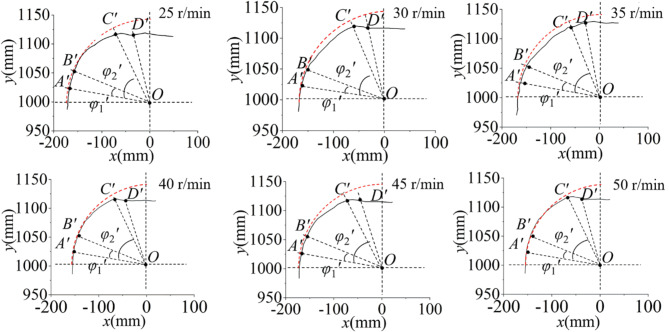
ESR process trajectory of seed potato in scenario 1.
  **Fig. 9:**
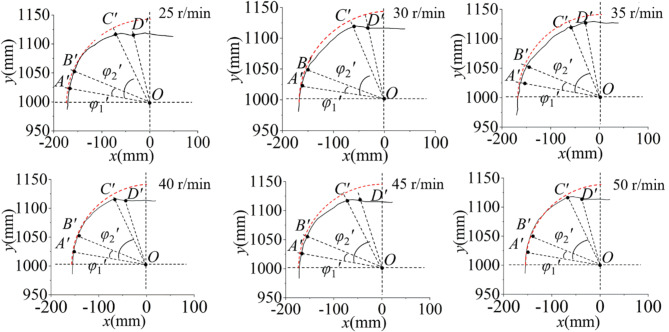
ESR process trajectory of seed potato in scenario 2.
  **Fig. 10:**
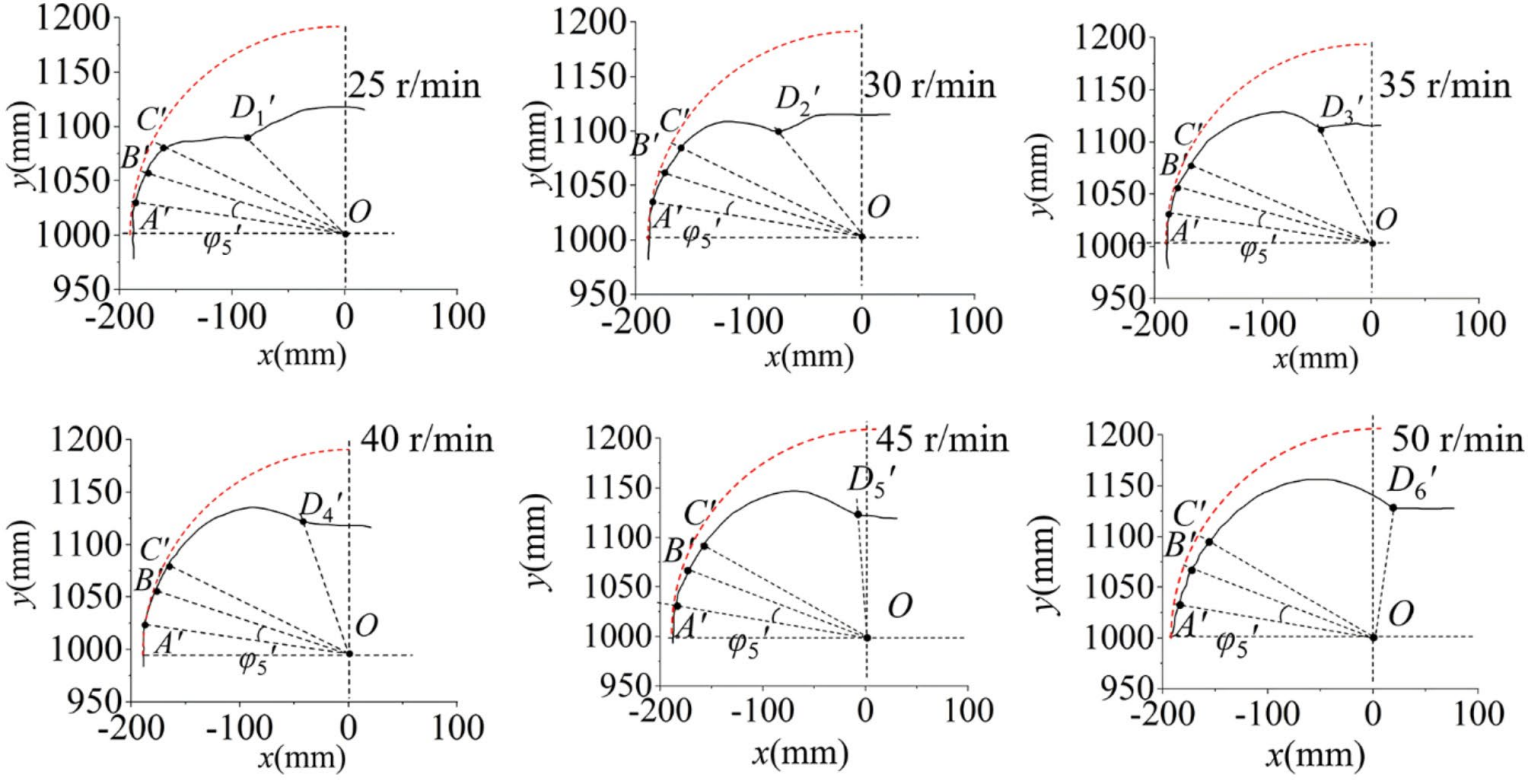
ESR process trajectory of seed potato in scenario 3.
2. Double seed collecting. The trajectories of ESR process in double SCSs under different sprocket rotational speeds were obtained as shown in Fig. 11. The dashed curve is the trajectory of seed potato without relative movements to the scoop during the simulation.
  **Fig. 11:**
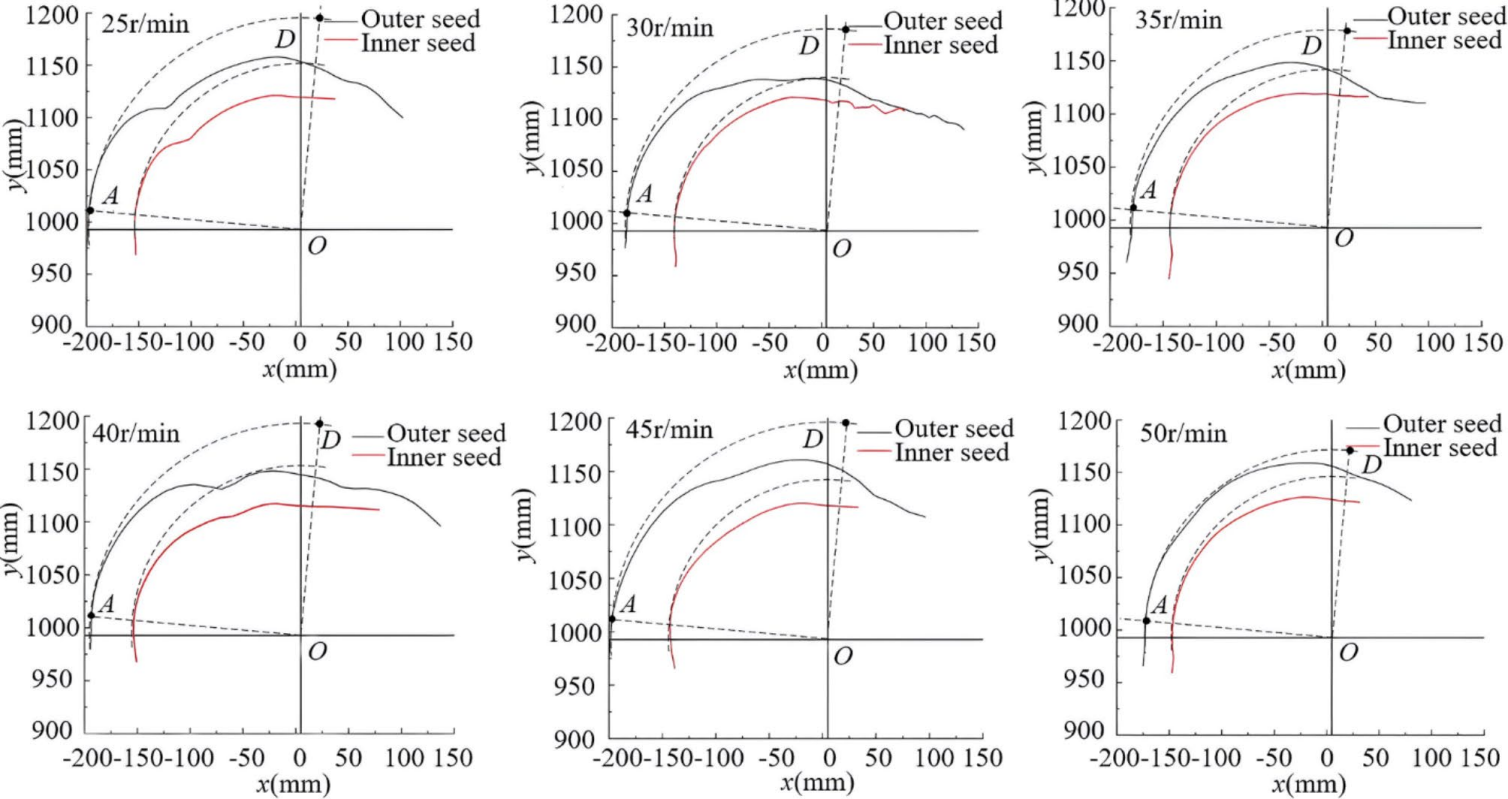
Trajectory of double seed removing.

### Bench and field tests

1. Bench test results. In this test, the collected data in the bench tests were statically recorded and analyzed. The average miss-collecting rate, average multi-collecting rate, average qualified ESR rate, average miss-seeding rate, average multi-seeding rate and average miss-seeding rate were recorded. The average value of these test results was calculated, as shown in Table 1. The Fig. 12 showed the variation of ESR performance with the sprocket rotational speed.
  **Table 1:** Bench test results.

Sprocket rotational speed	Testing groups			
		B1C3	B2C2	B3C1
A1	Average miss-collecting rate %	2.0	2.2	2.0
	Average multi-collecting rate %	54.8	39.6	23.6
	Average qualified-ESR rate %	86.6	95.8	92.0
	Average qualified-seeding rate %	86.2	91.6	92.2
	Average multi-seeding rate %	8.0	4.2	0.0
	Average miss-seeding rate %	5.8	4.2	7.8
A2	Average miss-collecting rate %	2	3.8	6.8
	Average multi-collecting rate %	44	32.8	22.8
	Average qualified-ESR rate %	92	85.8	86.6
	Average qualified-seeding rate %	88	88.4	89.6
	Average multi-seeding rate %	4	3.8	0
	Average miss-seeding rate %	8	7.8	10.4
A3	Average miss-collecting rate %	46.2	57.6	68.6
	Average multi-collecting rate %	2.4	8.4	9.8
	Average qualified-ESR rate %	90.4	81	81.8
	Average qualified-seeding rate %	83	86.4	82.4
	Average multi-seeding rate %	4.8	1.8	0
	Average miss-seeding rate %	12.2	11.8	17.6
  **Fig. 12:**
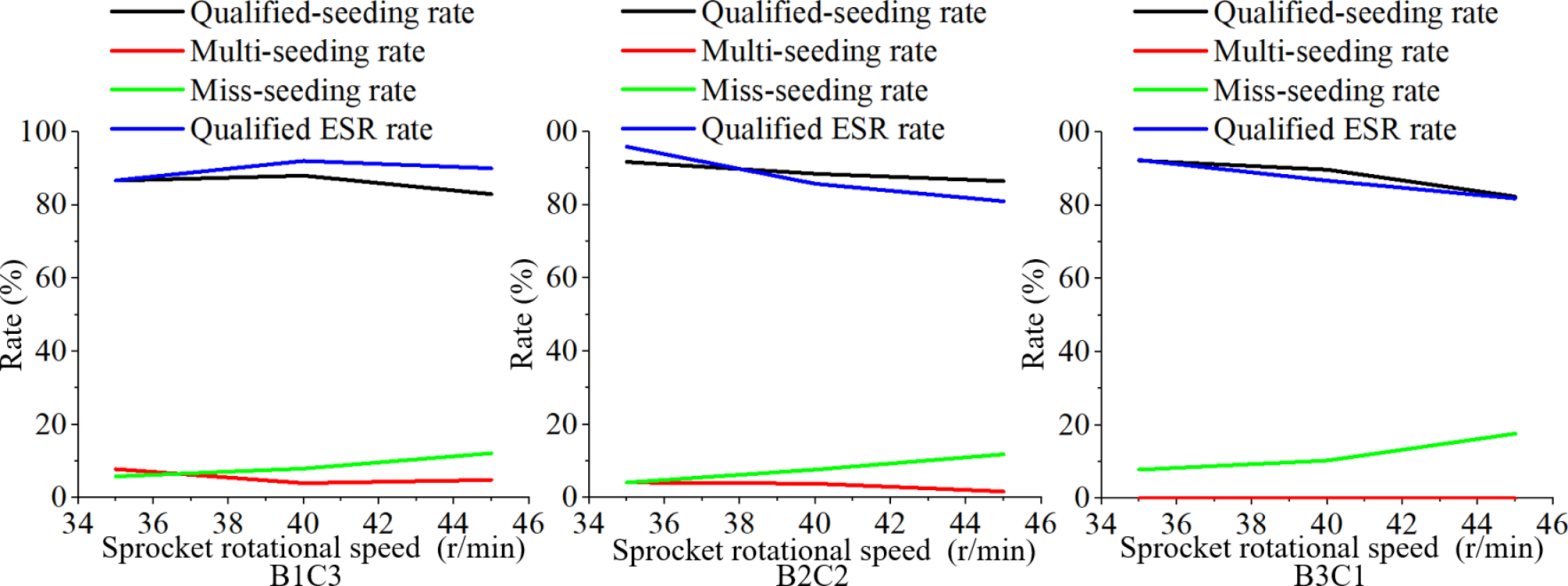
Relationship between indicators and sprocket rotational speed.
2. Field test results. We recorded the miss-seeding pits and multi-seeding pits among the 260 pits in every five test groups for analysis. The corresponding multi-seeding rate, miss-seeding rate and qualified-seeding (single) rate were calculated. The field test results are shown in Table 2.
  **Table 2:** Field test results.

Testing group	Total pit	Miss-seeding number	Multi-seeding number	Miss-seeding rate (%)	Multi-seeding rate (%)	Qualified-seeding rate (%)
1	260	11	15	4.23	5.77	90
2	260	9	16	3.46	6.15	90.38
3	260	12	15	4.62	5.77	89.62
4	260	10	13	3.85	5	91.15
5	260	8	14	3.08	5.38	91.54

## Analysis and discussion

### Numeric simulation

1. Single seed collection scenarios ESR results. The area between line *OA’* and *OB’*, *OB’* and *OC’*, *OC’* and *OD’* are few sliding area, sliding or parabolic area and main parabolic area in simulation, respectively. Since the selected simulation time is different in different sprocket rotational speed, the initial positions of seed potato trajectories are different. For the convenience of trajectory analysis, a quarter dashed circle concentric with the sprocket was drawn. The seed potatoes moved near the dashed curve, and the sliding movements in the process were few. Figure 8 showed the trajectories of seed potato’s centroid at different sprocket rotational speeds in scenario 1. In the few sliding area, the seed potatoes’ movement trajectories were similar and nearly parallel to the dashed curve line, indicated there were little sliding movements, and the angle *φ*_1*’*_ was about 25°. In the sliding or parabolic area, the trajectories deviated from the dashed curve line and shifted towards the center of the sprocket as the seeds moved toward the first seed collecting groove. Then, the trajectories were quickly turned to nearly horizontal after line *OC’*, demonstrated there were few parabolic or slipping movements, and the angle *φ*_2*’*_ was about 60°. In the main parabolic area, the seed potato fell into the first seed collecting scoop and moved nearly horizontally, and the ESR process was finished. We can see the influence of different sprocket speeds on the seed potato trajectory in scenario 1 was not significant, and the scenario 1 is stable. The Fig. 9 showed that the trajectories of seed potatoes’ centroid in scenario 2 at different sprocket rotational speeds. We can see the trajectories were nearly parallel to the dashed curve line in few sliding area, indicated there was few sliding movements, and the angle *φ*_3*’*_ was about 23°. In the sliding or parabolic area, the seed potatoes quickly moved into the first seed collecting groove due to the scoop flipping action and its own sliding movements, and the angle *φ*_4*’*_ was about 55°. The trajectories in main parabolic area were short and nearly parabolic. This indicated the scenario 2 is stable, and we can see the influence of different sprocket speeds on the seed potato trajectory in scenario 2 was not significant. Figure 10 showed the impact of sprocket rotational speeds on the seed potato’s trajectories in scenario 3, with the trajectories initially on the cushion and away from the seed collecting groove. Compared with the dashed curves in sliding area, the trajectories showed that there were nearly no sliding movements in different sprocket speeds, and *φ*_5*’*_ was about 18°. In the sliding or parabolic area, the trajectories changed, especially at 25 r/min speed, the seed potato moved into the groove in a circular motion. The actual end position of ESR process in scenario 3 under different sprocket rotational speed is different. Higher speeds resulted in more parabolic area and later end position. The line of *OD*_1*’*_, *OD*_2*’*_, *OD*_3*’*_, *OD*_4*’*_ and *OD*_5*’*_ represent the end line at the sprocket rotational speed of 25 r/min, 30 r/min, 35 r/min, 40 r/min, 45 r/min and 50 r/min. At the sprocket rotational speed of 25r/min to 45 r/min, the seed moved smoothly into the groove. We can see with the increase of sprocket rotational speed, the parabolic trajectory showed the seed potato was thrown higher, and the parabolic moving area was increased. But at 50 r/min, the trajectory showed the seed potato moved downward after the line *OD*_5*’*_, indicated the seed was thrown out of scoop, and caused irregular motion and collision with scoop edge. Thus, we can see the appropriate sprocket rotational speed for the ESR is 25 r/min-45 r/min. The seed potato in scenario 3 will be removed out of scoop and causing miss-seeding if the sprocket rotational speed is higher than 45 r/min. And we can see with the increase of sprocket rotational speed, the parabolic area of potato increased. Similarly, the seed potato in scenario 2 also can be removed out of scoop if the sprocket speed is too high. And the theoretical analysis in ESR of scenario 2 and 3 are verified.
2. Double seed collection scenarios ESR results. Initially, the centroid of the outer seed potatoes was higher than the inner seed potatoes. After the ESR process, the inner seed potatoes continued to move in a nearly horizontal line at the bottom of the collecting groove, with their trajectories gradually converging. The trajectories of outer seed potatoes were longer than inner seed potatoes and moved downward, indicated they were successfully removed out from the scoops. During the process, the space occupied by the inner seed potatoes in the groove limits the movement of the outer seed potatoes towards the inner circle. When the inner seed potatoes moved, the outer seed potatoes followed, and showed similarities in their trajectories. At low sprocket rotational speed, the inertia centrifugal force on the inner seed potatoes was lower than outer seed potatoes, leading to rapid changes in their movement trajectories. As the rotational speed increased, the inertia force increased, and the curve corner appeared closer to the end of the ESR process. At this point, the scoop was nearly vertical, with the inner seed potatoes restricted by the groove, allowed the outer seed potatoes to move in a parabolic trajectory. The outer seed potatoes rolled off from the inner seeds, the potential energy converted into kinetic energy, and resulted in a longer trajectory of outer seed potatoes.
  We can see the simulation results are coincident with the theoriotical analysis. During the flipping process to remove excess seeds, the cut potato slides into the collecting groove after achieving a sufficient angle. Scenarios 1 and 2 showd stable seed retention ability at the tested speeds, without being removed during the process. For scenario 3, a safe sprocket rotational speed is 25–45 r/min. The sprocket rotational speed of 25–50 r/min is suitable for ESR in double SCSs. The faster the sprocket rotational speed, the more seed potato can be removed out of the scoop.

### Bench and field tests

1. Bench test results. With the increase of sprocket rotational speed, the qualified-seeding rate in all experimental groups showed a downward trend, the miss-seeding rate gradually increased, the multi-seeding rate decreased. The qualified ESR rate in B1C3 firstly increased then decreased, and in B2C2 and B3C1 decreased. For the small seed potatoes and large pads group B1C3, the highest seeding performance was achieved at the sprocket rotational speed of 40 r/min, with highest qualified-seeding rate of 88%, multi-seeding rate of 4%, miss-seeding rate of 8.0% and qualified ESR rate of 92%. For the medium potato seeds and medium pad group B2C2, the suitable seeding performance was at 35 r/min, with qualified-seeding rate of 91.9%, multi-seeding rate of 4.2%, miss-seeding rate of 4.2%, and qualified ESR rate of 95.8%. In the large seed potatoes and small pad group B3C1, the best seeding performance was also at 35 r/min, with a qualified-seeding rate of 92.2%, multi-seeding rate of 0%, miss-seeding rate of 7.8%, and qualified ESR rate of 92%. Due to the low levels of double and multiple-seed collecting ratesand the unstable scenario of large potato seeds in the scoop, the kinetic energy of the upper layer of potato seeds was higher during ESR process, enhanced the seed flow. This resulted in a stable multi-seeding rate of 0 at increased sprocket rotational speeds due to the effective removing of double and multiple seeds. However, because of the strong ESR performance, some singly collected seeds were removed from the scoop during the ESR process and led to a high miss-seeding rate.
  The B1C3 group showed that, ESR process removed more seed potatoes, and higher sprocket speeds led to greater increases in miss-seeding rate. At 35 r/min, the increment of miss-seeding rate was the smallest but the multi-seeding rate was highest, indicated reduced ESR performance compared to 40 r/min. There was little difference between 40 r/min and 45 r/min in multi-seeding rate and multi-collecting rate changes, but miss-seeding rate increment at 45 r/min was significant, that is, high speeds will cause more seed potatoes fall out of scoop and high miss-seeding rate. Thus, 45 r/min is not suitable for seeding small seed potatoes. At 40r/min, ESR performance was stable with highest qualification rate. The B2C2 group showed that miss-seeding rate significantly increased with sprocket speed for medium seed potatoes. At 35 r/min, the difference of multi-collecting rate and multi-seeding rate was relatively big, indicated the ESR performance in this sprocket rotational speed was the strongest. Similarly, B3C1 group showed that, as sprocket rotational speed increased, miss-seeding increased significantly. At 40 r/min, miss-seeding rate increased compared to 35 r/min but multi-collecting rates didn’t change significantly. The appropriate speed for large seed potatoes was 35 r/min.
2. Field test results. According to Table 2, The Group 5 has the best seeding performance in the field tests with a qualified-seeding rate of 91.54%, miss-seeding rate of 3.08%, and multi-seeding rate of 5.38%. Other groups have similar results. The average rates across all four groups were 90.54% for qualified-seeding rate, 3.85% for miss-seeding rate, and 5.62% for multi-seeding rate. We can see the adaptability of the precision seed metering device to the cut potatoes is well and the seeding performance is good.
  From the theoretical analysis and experiments results, we can see the shapes of cut potatoes are crucial factors affecting the seeding performance of the seed metering device, especially the ESR performance. Thus, to maintain a good ESR performance, the cut potatoes should be properly cut and have a suitable size. Field and bench test results also indicated the uniformity of shape of cut potato directly affects the ESR performance.
  Shortcomings and future works: This study has some shortcomings, and future work can be developed from the following aspects. Optimize the structure of the seed scoop. The cushion block in the seed scoop has a direct impact on the seed collecting and ESR process. In subsequent research, we will analyze the effects of different buffer block structures on the seed extraction and cleaning processes, utilizing simulation software to simulate the seeding process. It is also necessary to establish a miss-seeding detection and automated reseeding system. To further achieve precise seeding of cut potato, research on potato miss-seeding detection technology should be conducted to analyze the accuracy of miss-seeding detection.

## Conclusions

In this study, a precision seed metering device and a novel scoop were introduced and analyzed. According to the number of seed potatoes in the scoop, the seed collecting was divided into three SCSs. The theoretical and simulation analysis on the ESR performance obtained that the influence of sprocket rotational speed and SCSs on the actual ESR performance was significant. Through the statics and kinematic analysis, the theoretical suitable sprocket rotational speed was determined to be 22.9 r/min to 42 r/min. It was found that the seed potato in SCS 1 is stable. The suitable sprocket rotational speed for scenarios 2 and 3 was found to be 25 r/min-45 r/min. The appropriate sprocket rotational speed for double SCSs to remove excess seeds was 25 r/min-50 r/min. With the increase of sprocket rotational speed, the number of seed potatoes remove out of scoop increased, the multi-seeding rate significantly decreased, the ESR performance of the device improved, but the high sprocket rotational speed removed too many seed potatoes and caused higher miss-seeding rate. The bench tests and field tests verified the actual performance of the precision seed metering device was good, and showed the appropriate sprocket rotational speed for medium and large seed potato was 35 r/min. The average rates of qualified-seeding rate, miss-seeding and multi-seeding in field tests were 90.54%, 3.85% and 5.62% respectively.

## Data Availability

The datasets used in this study are available from the corresponding author on reasonable request.
